# Impact of body condition on influenza A virus infection dynamics in mallards following a secondary exposure

**DOI:** 10.1371/journal.pone.0175757

**Published:** 2017-04-19

**Authors:** Nicholas G. Dannemiller, Colleen T. Webb, Kenneth R. Wilson, Kevin T. Bentler, Nicole L. Mooers, Jeremy W. Ellis, J. Jeffrey Root, Alan B. Franklin, Susan A. Shriner

**Affiliations:** 1 Department of Fish, Wildlife, and Conservation Biology, Colorado State University, Fort Collins, Colorado, United States of America; 2 United States Department of Agriculture, Animal and Plant Health Inspection Service, Wildlife Services, National Wildlife Research Center, Fort Collins, Colorado, United States of America; 3 Department of Biology, Colorado State University, Fort Collins, Colorado, United States of America; Centre for Ecology and Evolution in Microbial Model Systems, SWEDEN

## Abstract

Migratory waterfowl are often viewed as vehicles for the global spread of influenza A viruses (IAVs), with mallards (*Anas platyrhynchos*) implicated as particularly important reservoir hosts. The physical demands and energetic costs of migration have been shown to influence birds’ body condition; poorer body condition may suppress immune function and affect the course of IAV infection. Our study evaluated the impact of body condition on immune function and viral shedding dynamics in mallards naturally exposed to an H9 IAV, and then secondarily exposed to an H4N6 IAV. Mallards were divided into three treatment groups of 10 birds per group, with each bird’s body condition manipulated as a function of body weight by restricting food availability to achieve either a -10%, -20%, or control body weight class. We found that mallards exhibit moderate heterosubtypic immunity against an H4N6 IAV infection after an infection from an H9 IAV, and that body condition did not have an impact on shedding dynamics in response to a secondary exposure. Furthermore, body condition did not affect aspects of the innate and adaptive immune system, including the acute phase protein haptoglobin, heterophil/lymphocyte ratios, and antibody production. Contrary to recently proposed hypotheses and some experimental evidence, our data do not support relationships between body condition, infection and immunocompetence following a second exposure to IAV in mallards. Consequently, while annual migration may be a driver in the maintenance and spread of IAVs, the energetic demands of migration may not affect susceptibility in mallards.

## Introduction

Influenza A viruses (family Orthomyxoviridae, genus *Influenzavirus A*) present numerous challenges for both human and animal health, including major economic and disease burdens [1]. Waterfowl and shorebirds of the orders Anseriformes (e.g. ducks, geese, and swans) and Charadriiformes (e.g. gulls, terns, and shorebirds) serve as natural reservoirs for influenza A viruses (IAVs), contributing to their spread and maintenance within the environment [2]. In particular, dabbling ducks within the genus *Anas* are regarded as primary reservoir hosts [3]. While peak prevalence in the Northern hemisphere generally occurs in these birds when immunologically-naïve, juvenile birds are exposed to IAVs at congregation sites prior to and during fall migration, infections can occur year-round [4, 5].

Given the diversity of IAVs, antigenically distinct subtypes co-circulate within natural populations over temporal and spatial scales, and individuals can be re-infected by the same subtype (i.e. homologous infection) or infected by a different subtype altogether (i.e. heterologous infection). Previous studies addressing serial infections have reported variable immunity to reinfection with low pathogenic (LP) IAVs in domestic and wild birds [6–8]. Consequently, the co-circulation of LPIAV subtypes, compounded with varying homosubtypic and heterosubtypic protection, creates a complex immunity landscape [9], which needs to be further explored in order to gain a holistic understanding of IAV infection dynamics.

Mallards (*Anas platyrhynchos*) naturally infected with an IAV were observed to have lower body mass than non-infected mallards [10]. Although only in specific years, a similar result was found in greater white-fronted geese (*Anser albifrons*) that were also naturally infected with an IAV [11]. However, because these were observational studies on free-ranging birds, causation between body condition and infection with a IAV is difficult to conclude [12]. As a result, an alternative explanation has been developed, suggesting that individuals in poorer body condition have reduced immunocompetence and are more susceptible to IAVs (i.e. “the condition-dependent hypothesis”) [13].

Recent examination of the condition-dependent hypothesis in wild populations showed only minor differences in body conditions between infected and uninfected wild mallards [14]. This result runs contrary to the proposed hypothesis and may be due to differences in the timing of sample collection [14]. In the previous study, birds were sampled during migration at stopover sites where a large proportion may still have been naïve and experiencing a primary infection [10]. In contrast, in the more recent study, birds were sampled on their wintering grounds when most individuals may have been previously infected and be partially protected by heterosubtypic immunity as a result [14]. However, after conducting an experimental inoculation with LPIAV in wild-caught mallards, it was found that susceptibility to infection and viral excretion were lower in individuals with lower body conditions scores [15]. This result is also in contrast to the condition-dependent hypothesis, and suggests that birds in poorer body condition are less susceptible and competent to infection with the tested IAV strain.

Our study tested the impact of body condition on the infection dynamics of captive mallards challenged with an IAV using methods similar to those previously published [15]. In our study, however, the challenge was a secondary exposure after a natural infection with a heterosubtypic virus; consequently, we assessed infection dynamics and immune response as a function of body condition for a heterosubtypic serial infection. Overall, the goal of our study was to experimentally assess the relationship between body condition and infection with IAVs.

## Material and methods

### Ethics

All experiments were approved by the Institutional Animal Care and Use Committee of the United States Department of Agriculture (USDA), Animal and Plant Health Inspection Service (APHIS), Wildlife Services, National Wildlife Research Center (NWRC, Approval 2385), Fort Collins, CO, USA.

### Animals & housing

Thirty hatch-year mallards (15 males and 15 females) were randomly selected from a captive research flock held in an outdoor flight pen. The flock originated from a group of mallards purchased from Murray McMurray Hatchery (Webster City, IA) in February 2012. Some of the purchased ducks were allowed to breed in 2014 and their offspring were used in the current study. Duck ages ranged from 27–33 weeks at inoculation. Sentinel mallards in the flight pen were screened for exposure to IAV every four weeks. Ten mallards were randomly assigned to each of the three *a priori* treatment groups (control, -10% body weight, and -20% body weight). Prior to the onset of the study, blood was collected from each mallard in order to obtain baseline immune function data. Mallards were serologically tested for exposure to IAV prior to diet manipulation to ensure they were immunologically naïve. During diet manipulation, mallards were individually housed in screened pens enclosed within a large outdoor screened and covered shelter. Pens were equipped with a shallow water bowl, food bowl, and an artificial pond. During the experimental inoculation, mallards were individually housed in stainless steel animal racks within a biosafety level 2 (BSL-2) animal room. LPIAV is not generally associated with any observable clinical symptoms. All individuals were inspected at least once a day for signs of pain or distress. While no clinical signs or mortalities were observed throughout the study, the protocol for distressed animals (e.g. those exhibiting lethargy, diarrhea, emaciation, recumbency) would have been either 1) treated as directed by the attending veterinarian and retained in the study or 2) euthanized.

### Diet manipulation

Mallard baseline food consumption was identified during the first week of the study followed by five weeks of provisioning, with a daily food ration consistent with its assigned treatment group. Body weights were recorded each weekday (Monday-Friday) to assess body condition. Control birds were fed *ad libitum*, and the -10% and -20% body weight groups were given approximately 85% and 75% of the baseline food consumption, respectively. Any bird that was not trending toward its assigned body condition group had food rations further restricted; similarly, any bird that lost weight too fast (>5% body weight loss per day) or exceeded its body condition group goal was given increased food rations. At the end of this phase, birds were once again tested for exposure to IAV.

### Natural exposure

ELISA results from sera collected after the diet manipulation on day 0 of the experimental inoculation indicated that all 30 study subjects were naturally exposed to IAV prior to inoculation. Testing of sentinel ducks from the outdoor flight pens where the research flock was held indicated the timing of the exposure was likely immediately prior to the movement of mallards to individual pens for diet manipulation. Ducks were bled the day they were moved, but were seronegative, likely because they were in an early stage of infection prior to the mounting of a detectable humoral antibody response (usually detectable within 4–5 days post infection for a primary IAV infection). Serum was submitted to the National Veterinary Services Laboratory (Ames, IA) for hemagglutinin inhibition testing and exposure to an H9 IAV was confirmed. As a result of this natural exposure in the study ducks, the experiment does not include a naive control group, but rather, all three treatment groups experienced a prior natural exposure to an H9 virus approximately seven weeks before the H4N6 inoculation.

### Experimental virus and inoculation

At the end of the diet manipulation, mallards were experimentally infected with an avian LPIAV [A/mallard/CO/P66F1-5/08 (H4N6)], originally collected from a wild bird as part of a U.S. national surveillance system for avian influenza initiated in 2006 [16] and passaged through a mallard prior to virus isolation in chicken eggs. This virus was selected because it is among the most commonly isolated subtypes from North American waterfowl [17, 18]. Virus stocks were propagated and passaged using methods described previously in detail [19]. Viral titers were calculated using the Reed and Muench method [20]. All 30 mallards were orally inoculated twice (with approximately four hours between inoculations) with 1mL of diluted IAV (H4N6) containing approximately 10^4^ EID_50_ virions. Body condition classes were maintained during the experimental infection period by continuing to restrict food rations based on daily body weight measurements. On 1–7, 10, and 14 days post inoculation (DPI), each bird was sampled by collecting an oral, cloacal, and fecal swab. Swabs were placed in 1mL of BA-1 viral transport media [21] and stored at 80°C until testing. In addition, blood samples were collected and two duplicate blood smears were made per mallard on 0, 2, 4, 7, 10, and 14 DPI. At 14 DPI, all ducks were euthanized.

### Prior experimental inoculation of naïve mallards

In order to quantify the potential impact of heterosubtypic immunity gained from the H9 exposure on viral output from the H4N6 infection, we used data available from a similar, previously published study [22]. We compared H4N6 excretion patterns from that study’s experimentally infected naïve ducks to the H4N6 excretion from the ducks in our study that had been exposed to an H9 virus. In the earlier study, 15 naïve mallards fed an *ad libitum* diet were inoculated with 1mL of the same diluted H4N6 virus stock used in the current study. Briefly, mallards were inoculated with 1mL of diluted IAV (H4N6) containing approximately 10^5^ EID_50_/mL (compared to 2mL of 10^4^ EID_50_/mL used in the current study). Mallards were housed in a biosafety level 2 animal room with 3 mallards/pen (compared to individually housed birds in cages in the current study). Duck ages, room conditions (i.e. temperature and light regime), diets, oral and cloacal sample collection, and sample analysis followed the same protocols for both studies.

### Viral detection and quantification

Oral, fecal, and cloacal swabs were tested by quantitative, real-time, reverse transcriptase polymerase chain reaction (qRT-PCR). Viral RNA was extracted using MagMax-96 AI/ND Viral RNA Isolation Kits (Thermo Fisher Scientific, Inc., Waltham, MA). RNA extracts were tested in duplicate using primers and a probe specific for the influenza type A matrix gene previously described in the literature [23] using CFX96 Touch Thermocyclers (Bio-Rad Laboratories, Inc., Hercules, CA). Thermocycler conditions previously described were utilized [24], with the exception that plates ran for 40 cycles of 95°C for 15 sec and 60°C for 30 sec. Calibrated controls with known viral titers (10^2^ EID_50_/mL–10^5^ EID_50_/mL) were analyzed in duplicate to construct four-point standard curves. Sample viral RNA quantities were extrapolated from the standard curves and are reported as PCR EID_50_ equivalents/mL. Cycle quantities (Cq) were standardized by setting the baseline to a uniform threshold for all runs.

### Serology and immune assays

Serum samples collected on 0, 2, 4, 7, 10, and 14 DPI were tested for IAV antibodies via the FlockCheck Avian Influenza MultiS-Screen Antibody Test Kit (IDEXX Laboratories, Inc., Westbrook, ME), following the manufacturer’s instructions. Experimental testing of wild birds using this ELISA kit has shown that an alternative threshold of 0.7 optimizes correct classifications (i.e. seropositive or seronegative), as opposed to the threshold of 0.5 suggested by the assay manufacturer [25, 26]. Therefore, we applied a threshold S/N ratio <0.7 to identify positive samples. Haptoglobin concentrations (mg/mL) for the pre-bleed serum samples prior to diet manipulation as well as the 0 and 4 DPI serum samples were quantified using a commercially available assay, following the packet insert (no. TP-801; Tridelta Development Ltd., Maynooth, Ireland). From the two blood smears per mallard collected on 0, 2, 4, 7, 10, and 14 DPI, the first 100 leukocytes visualized on the first smear were classified and enumerated (the second was used as a back-up if the first slide was of low quality). Based on these counts, the ratio between heterophils and lymphocytes (H/L) was calculated.

### Statistical analysis

We compared the three body condition classes for each of the viral and immune function variables measured (i.e., total viral RNA detection for oral, cloacal, and fecal swabs, antibody signal, haptoglobin levels, and heterophil/lymphocyte ratios) using mixed effect regression models with individuals as a random effect. For viral output, the model tested total viral RNA output as a function of body condition class, swab type, and sex. For the immune function variables, a repeated measures regression was used and the model tested each immune factor as a function of body condition class, day post inoculation, and sex. All models were implemented in Program R (R Development Core Team, Vienna, Austria) version 3.2.3, using the ‘lme4’ package [27].

## Results

**Fig 1:**
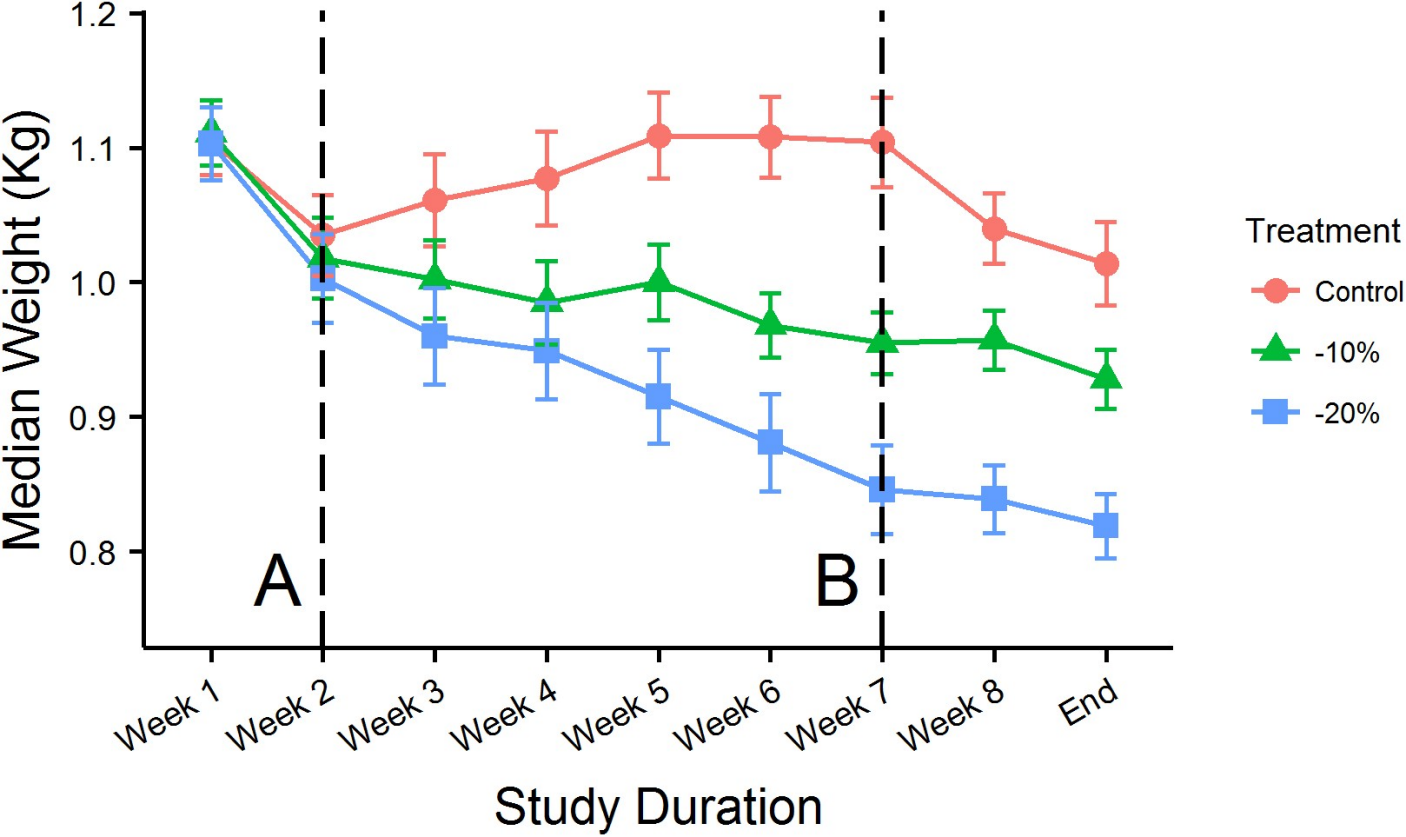
Median Treatment Weights. The median weights of each treatment group every week over the duration of the study. Error bars show +/- one standard error for each respective day. Line A denotes the beginning of diet manipulation and Line B denotes when the birds were experimentally inoculated.

Diet manipulation separated the three treatment groups after five weeks of controlled intake (Fig 1). When compared to the control treatment group, median weight loss prior to the inoculation was -10.54% (SD = 1.79%) in the -10% treatment group, and -20.68% (SD = 6.92%) in the -20% treatment group. The initial drop in weights in week 1 was associated with moving the birds from a large outdoor flight pen to individual outdoor pens. A second dip from after the inoculation, primarily seen in the control group, was likely associated with the move from the individual outdoor pens into stainless steel cages in the BSL-2 facility.

### Heterosubtypic response to H9 infection

**Fig 2:**
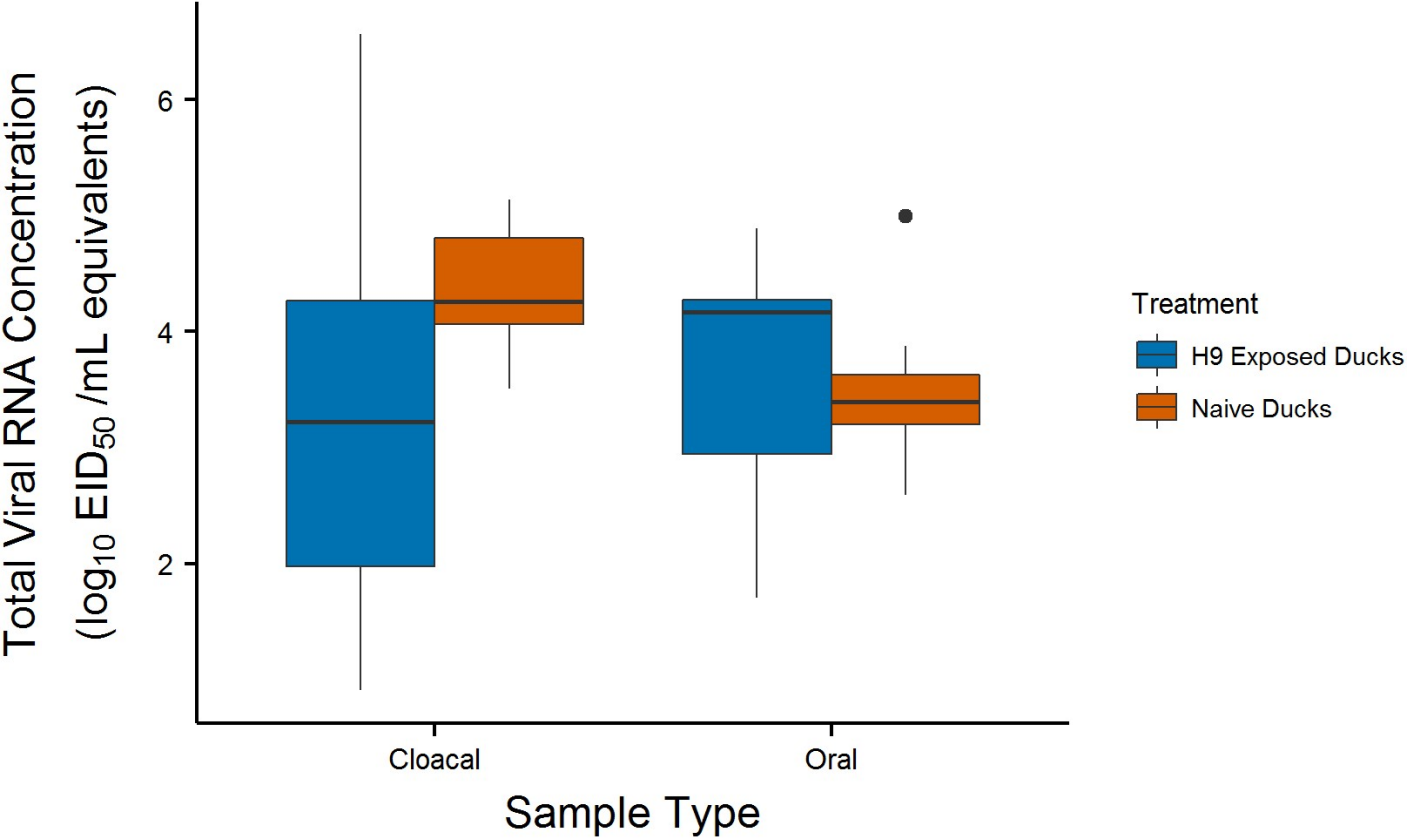
Heterosubtypic Immunity. Box plots summarize the distribution of total viral shedding in oral/cloacal swabs taken from the H9-exposed control group and naïve ducks from another study [22]. Boxes show the interquartile range (IQR, middle 50% of values) for each group, horizontal lines represent medians, and vertical lines are values within 1.5*IQR. Outliers are plotted as individual points.

To infer the degree of immunity the birds gained in response to the H9 natural exposure, we compared total viral output between the control body condition group from the current study to naïve ducks from a previously published study that were inoculated with the same H4N6 virus (Fig 2)[22]. Naïve ducks shed longer compared to the control ducks naturally exposed to H9 for cloacal swabs, but viral RNA output duration was similar for oral swabs. For cloacal swabs, 10 of 15 naïve ducks had cloacal viral RNA concentrations greater than 10^1^ EID_50_/mL on 7 DPI compared to 1 of 10 H9-exposed ducks. Similarly, naïve ducks exhibited median peak shedding (cloacal sample range = 3.38–4.96 log_10_ EID_50_/mL equivalents for naïve ducks and 0–4.74 log_10_ EID_50_/mL equivalents for H9-exposed ducks), but not for oral swabs. The increased oral RNA excretion for the H9-exposed ducks may have been due to the dual inoculation method for those ducks (compared with a single inoculation for the naïve ducks) in which we inoculated once in the morning and then again in the afternoon. Because of this methodological difference between the two studies, caution must be applied in interpreting these results.

### Peak viral load and duration of viral detection

**Fig 3:**
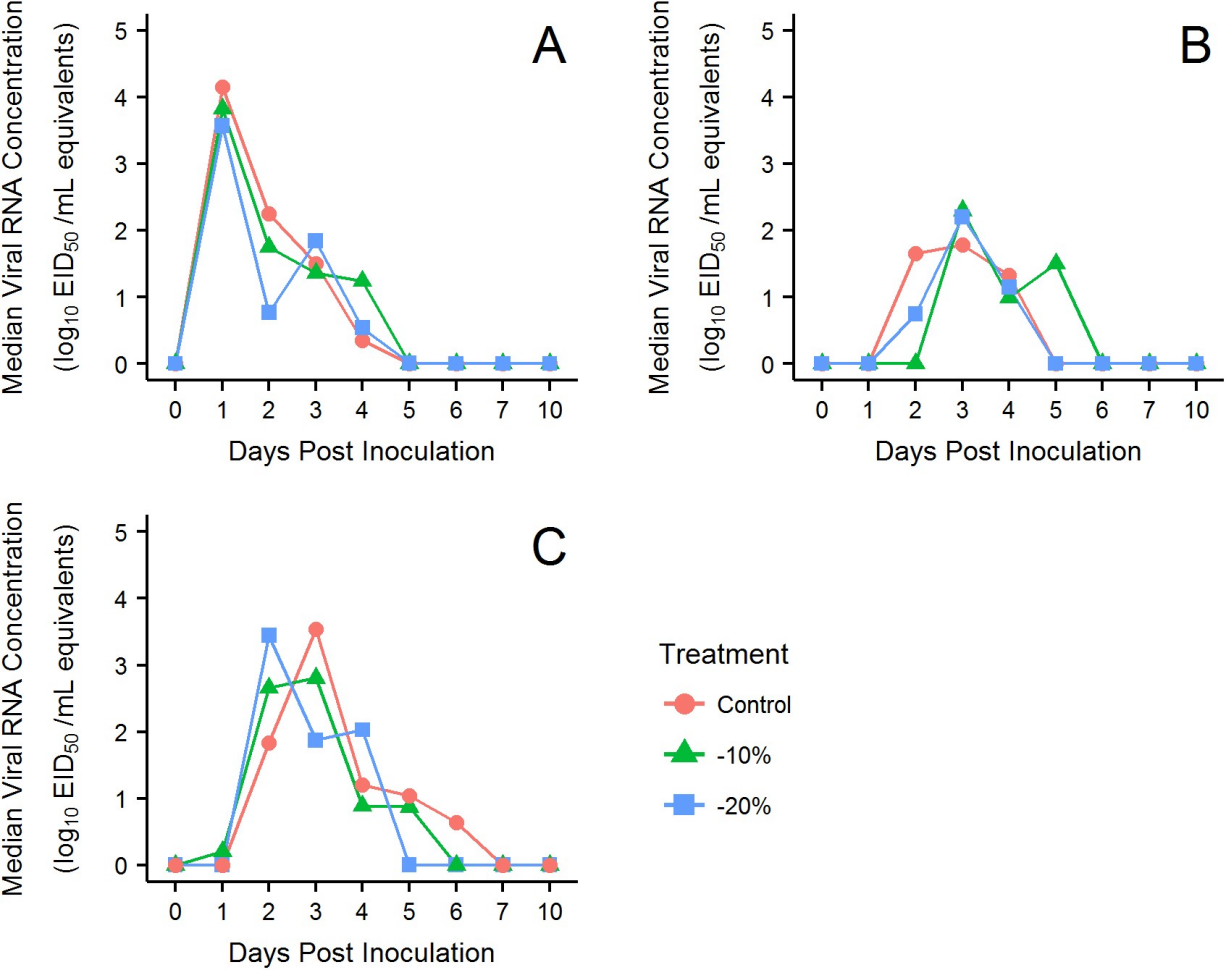
PCR Results. Line plots of median viral RNA concentrations. The areas under the curves are indexes for the total amount of virus detected across the infection. (A) oral swabs, (B) cloacal swabs, and (C) fecal swabs.

The median peak oral viral RNA concentration (log_10_ EID_50_/mL equivalents) detected for all treatment groups occurred at 1 DPI (control SD = 0.8 DPI, -10% SD = 0.3 DPI, -20% SD = 1.3 DPI). Median cloacal peak viral load for all treatment groups occurred at 3 DPI (control SD = 1.1 DPI, -10% SD = 1.2 DPI, -20% SD = 1.4 DPI). Median fecal peak viral loads occurred on 3 DPI for the control and -20% groups (control SD = 1.6 DPI and -20% SD = 1.7 DPI), and on 2 DPI (SD = 2.0 DPI) for the -10% group. The median duration that viral RNA was detected from oral swabs was 2 days (SD = 0.9 days) for the control group, and 4 days for the -10% and -20% groups (-10% SD = 1.6 days and -20% SD = 2.0 days). Median duration for cloacal viral shedding was 3 days for all treatment groups (control SD = 2.0 days, -10% SD = 1.1 days, -20% SD = 2.0 days). Fecal viral shedding lasted on average for 3 days (SD = 2.8 days) for the -10% body condition class and 4 days for the control and -20% classes (control SD = 2.8 days and -20% SD = 3.0 days). Overall, the median concentration of viral shedding did not significantly vary between treatment groups (p-value = 0.994; Fig 3).

### Serology, haptoglobin, and H/L ratios

**Fig 4:**
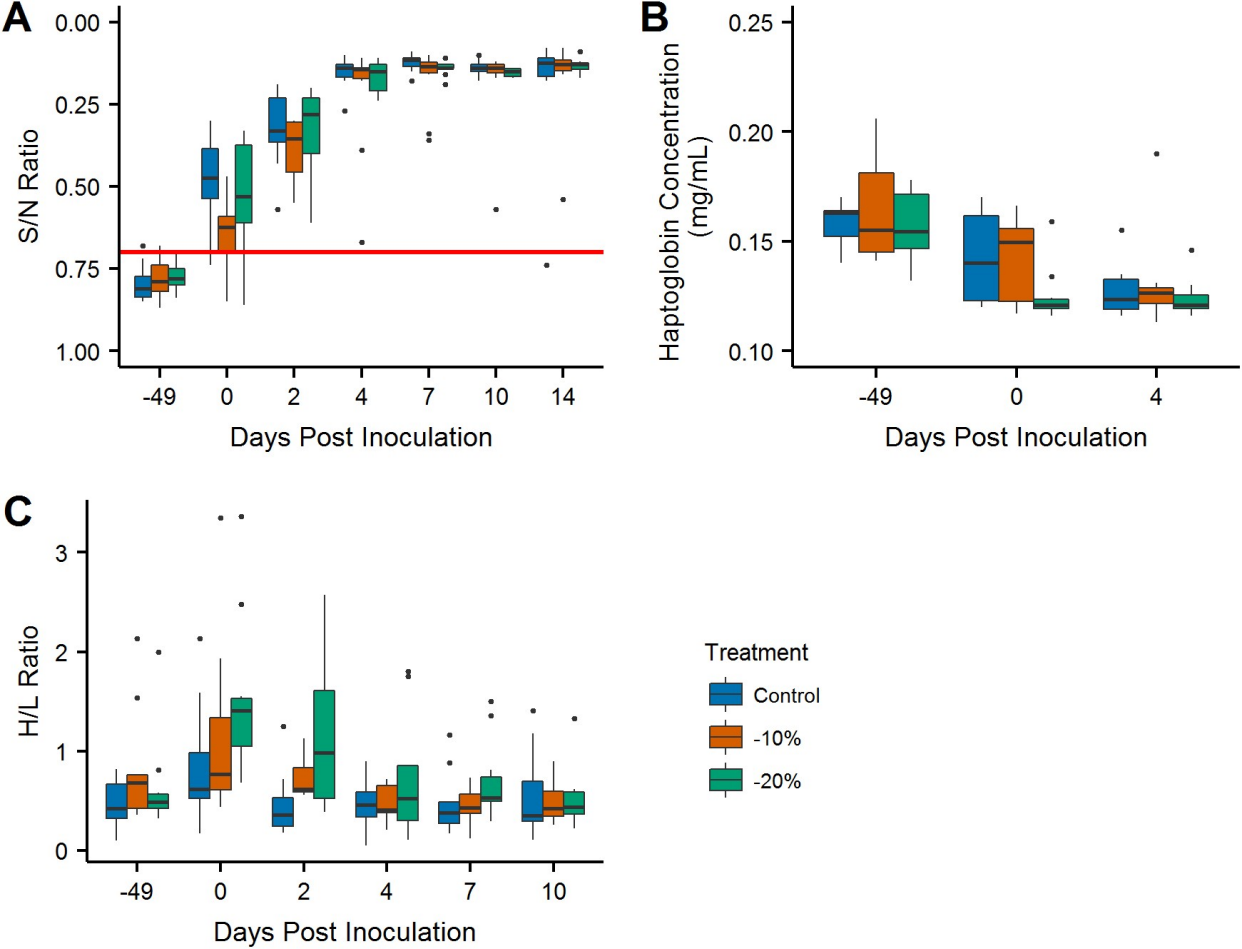
Immune Function. (A) Boxplots of the interpolated S/N ratios over the duration of the study. Values above the threshold S/N ratio of 0.7 (the red line) are considered positive for antibodies to IAV. (B) Boxplots of haptoglobin concentrations over the duration of the study. (C) Boxplots of heterophil/lymphocyte ratios over the duration of the study.

All birds were seronegative for antibodies to IAV before the diet manipulation phase, but were found to be seropositive or suspect positive on 0 DPI. A steep spike in median sample to negative (S/N) ratios on 2 and 4 DPI show a strong anamnestic response for the secondary H4N6 infection. All birds remained seropositive during the duration of viral shedding following 0 DPI (Fig 4A). Overall antibody production and S/N ratios did not significantly differ across treatment groups (-10% p-value = 0.079 and -20% p-value = 0.852). Haptoglobin concentrations were markedly lower on 0 and 4 DPI compared to the serum samples collected prior to the first week of the study across all three groups, but overall there was no significant variation between groups (Fig 4B; -10% p-value = 0.509 and -20% p-value = 0.076). H/L ratios were significantly higher for the -10% and -20% treatment groups (p-value = 0.046), and generally declined over time (Fig 4C).

## Discussion

Across all three treatment groups, mallards exhibited a similar anamnestic response to a heterologous infection with an H4N6 IAV following a natural exposure to an H9 IAV. This result suggests that exposure to an H9 subtype confers heterosubtypic immunity in mallards when challenged with an H4 subtype. While serial infection occurred over a relatively short duration (approximately 7 weeks), the timing of infections is consistent with what might occur naturally, given the diversity and prevalence of LPIAVs circulating during migration. Body condition class did not have a significant impact on peak antibody levels, although S/N ratios appeared to decrease more quickly in *ad libitum* fed ducks. All three treatment groups exhibited a rapid production of antibodies following the heterologous infection and reached similar peak antibody levels, suggesting body condition did not appear to impact the adaptive immune response. Given the significant individual heterogeneity found in antibody titers [28] and our limited samples sizes, this lack of significance between treatment groups may be due to inadequate statistical power. It is also possible that heterosubtypic immunity may have compensated for any effect reduced body condition may have on an individual. Finally, the secondary infection occurred over a short time span. IAV antibody titers in mallards generally do not begin to decline until about eight weeks post-infection, so circulating antibodies from the H9 infection most likely contributed to the observed S/N ratios, particularly at the beginning of the infections.

Haptoglobin is an acute phase protein that binds free hemoglobin, preventing it from providing nutrients to pathogens. Concentrations of haptoglobin and other acute phase proteins typically increase by 25% during an innate immune response to acute infection, inflammation, or trauma [29]. In a study of IAV in mallards, haptoglobin concentrations were found to be higher in infected juveniles and females compared to non-infected juveniles and females, but lower in infected adults and males compared to non-infected adults and males [14]. In our study, haptoglobin concentrations increased on 0 and 4 DPI for all three treatment groups, but overall there was no significant variation between males and females or treatment groups, suggesting that body condition did not impact this component of the innate immune response. Because concentrations also increased in the *ad libitum* ducks, we hypothesize that the increase in haptoglobin levels may have been associated with experimental housing conditions. Heterophils and lymphocytes are the two dominant leukocytes that mediate innate and adaptive immune responses, respectively. While an increase in the heterophils to lymphocytes (H/L) ratio can reflect stress and susceptibility to infection [30], it has been shown that feed restriction leads to increases in H/L ratios in chickens [30] and it is reasonable to assume a similar effect would be seen in mallards. This finding may explain why H/L ratios in both reduced body condition groups were significantly higher than the control group.

Birds in poor body condition did not excrete lower quantities of viral RNA or have a lower peak viral load as was found in a similar experiment that tested the impact of body condition for a primary exposure [15]. Instead, mallards in the restricted feed groups in our experiment excreted similar amounts of virus for the same duration as the control group. One potential factor in our study that may have increased heterogeneity in viral output and thereby reduced our statistical power to detect differences is that the age of the ducks varied from 27–34 weeks of age at inoculation. Some studies have shown that viral excretion can vary with age [31, 32], but limited data are available for ducks in the age range tested in our study. While we randomly assigned ducks to treatment groups to balance the age structure of the groups (the mean age differences between the three groups was less than a week), any heterogeneity associated with age differences may have reduced statistical power. Although our results run counter to the previous experimental evaluation of body condition for a primary IAV infection [16], our experimental results corroborate a large-scale field test [14] which also found no association between viral shedding and body condition. Given that we evaluated the impact of body condition for a secondary IAV exposure, our results are likely more similar to these field results since the ubiquity of IAVs during fall migration makes it likely that many wild bird exposures are secondary infections. Previous research has also shown that total viral output is negatively related to body condition [10], but our data does not support this.

Ultimately, the lack of differences in antibody production, haptoglobin concentration, and viral shedding between treatment groups do not replicate or validate previous results. Recent work in the emerging field of ecological immunology [33] suggests that nutritional condition is critical in mounting an immune response [34, 35] and that susceptibility is higher and infection intensity is more severe in individuals in poorer condition [36]. Despite these over-arching correlations and the conventional-wisdom that individuals in poor health bear a heavier infection burden [12], our study found that IAV infection dynamics in mallards in poorer body condition do not appear to differ from individuals in better condition. As such, the condition-dependent hypothesis should not be taken as universally applicable. These results lend credence to the conclusion that we do not fully understand the relationship between body condition and IAV infection dynamics, and a general model of this process has yet to be described. Although our diet manipulation does not fully mimic the toll of migration, it suggests that migration may not alter IAV infection dynamics, which leads to a relevant corollary: does IAV infection alter migration? While it has been suggested that migratory, wild birds do not spread highly pathogenic IAV because of negative effects on migratory performance due to infection [37], current field research does not support the idea that LPIAV infection has an impact the timing of or movements during migration [38, 39]. In tandem, the hypotheses that migratory body condition does not alter infection dynamics and infection does not modify migration underscore that migratory waterfowl may be an ideal vehicle for the spread of IAVs across large-geographic migration pathways.

An important aspect of appropriately interpreting the results presented in this study is to consider the potential impact of experimental limitations compared to natural systems. To start, no data are available on typical virus concentrations associated with natural transmission. Although the primary infection in this study was a natural exposure, the secondary infection resulted from an experimental inoculation with a controlled dose. If exposure dose has a strong impact on infection dynamics and our inoculating concentration was atypical of those experienced by natural populations, our results may not be representative of wild populations. A second consideration is whether our ability to manipulate body condition was a reasonable proxy for poor body condition in wild birds. The food restricted birds in our study as well as those in the previous experimental study [15] did not show a difference in body condition before or after inoculation. These results are consistent with previous experimental research evaluating clinical signs in response to LPIAV infection [40] which did not find an association between infection and body weight. In contrast, the large-scale field study that examined correlations between body weight and infection status found that IAV infection was associated with reduced body condition [10]. A final consideration is that we did not have an uninfected control group such that we are not able to fully disentangle changes in blood parameters associated with infection from those associated with body condition.

In conclusion, while previous research has demonstrated that differential body conditions impact shedding characteristics after a primary infection, our results show muted differences after a secondary, heterologous serial infection and do not support relationships between body condition, infection and immunocompetence. More experimental research is needed to determine whether our results are typical since IAV shedding dynamics vary significantly across strains. Future directions should also include validating whether this evidence holds for other species of waterfowl or shorebirds, and quantifying possible differences in aspects of the immune system we did not address. Although IAV infection in mallards typically lacks clinical signs and our data shows that body condition does not alter infection, we are guardedly skeptical that IAVs have become obligate commensals. Instead, it is probable that the virulence of IAVs manifests in subtle life-history trade-offs that should be explored within an ecological context such as migration or reproduction, not only to better understand host competence, but also because it is a compelling paradigm of virulence evolution.
